# *Streptococcus agalactiae* Infects Glial Cells and Invades the Central Nervous System *via* the Olfactory and Trigeminal Nerves

**DOI:** 10.3389/fcimb.2022.793416

**Published:** 2022-02-24

**Authors:** Anu Chacko, Ali Delbaz, Indra N. Choudhury, Tanja Eindorf, Megha Shah, Christopher Godfrey, Matthew J. Sullivan, James A. St John, Glen C. Ulett, Jenny A. K. Ekberg

**Affiliations:** ^1^Menzies Health Institute Queensland and School of Pharmacy and Medical Sciences, Griffith University, Southport, QLD, Australia; ^2^Clem Jones Centre for Neurobiology and Stem Cell Research, Griffith University, Nathan, QLD, Australia; ^3^Griffith Institute for Drug Discovery, Griffith University, Nathan, QLD, Australia

**Keywords:** *Streptococcus agalactiae*, peripheral nerve, bacteria, olfactory ensheathing cell, Schwann cell, astrocyte, central nervous system

## Abstract

*Streptococcus agalactiae* causes neonatal meningitis and can also infect the adult central nervous system (CNS). *S. agalactiae* can cross the blood-brain barrier but may also reach the CNS *via* other paths. Several species of bacteria can directly invade the CNS *via* the olfactory and trigeminal nerves, which extend between the nasal cavity and brain and injury to the nasal epithelium can increase the risk/severity of infection. Preterm birth is associated with increased risk of *S. agalactiae* infection and with nasogastric tube feeding. The tubes, also used in adults, can cause nasal injuries and may be contaminated with bacteria, including *S. agalactiae*. We here investigated whether *S. agalactiae* could invade the CNS after intranasal inoculation in mice. *S. agalactiae* rapidly infected the olfactory nerve and brain. Methimazole-mediated model of nasal epithelial injury led to increased bacterial load in these tissues, as well as trigeminal nerve infection. *S. agalactiae* infected and survived intracellularly in cultured olfactory/trigeminal nerve- and brain-derived glia, resulting in cytokine production, with some differences between glial types. Furthermore, a non-capsulated *S. agalactiae* was used to understand the role of capsule on glial cells interaction. Interestingly, we found that the *S. agalactiae* capsule significantly altered cytokine and chemokine responses and affected intracellular survival in trigeminal glia. In summary, this study shows that *S. agalactiae* can infect the CNS *via* the nose-to-brain path with increased load after epithelial injury, and that the bacteria can survive in glia.

## Introduction

**Figure 1:**
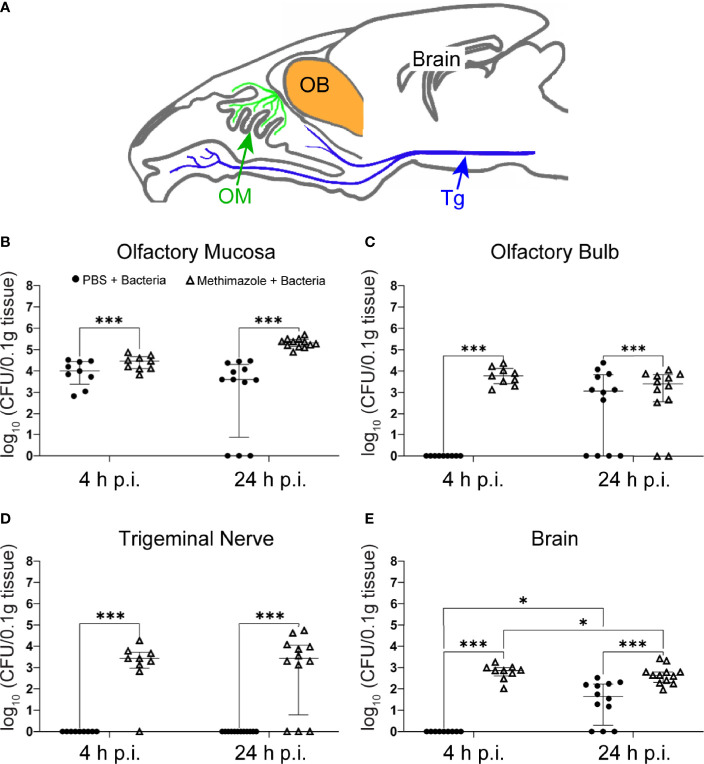
*S. agalactiae* can invade the CNS *via* the olfactory nerve but infects the trigeminal nerve only after epithelial injury. Mice were treated with either methimazole or vehicle (PBS), followed by intranasal inoculation with *S. agalactiae* (1 x 10^6^ CFU) or vehicle (also PBS). **(A)** Schematic of sagittal view of mouse brain, with olfactory mucosa (OM) and olfactory nerve (green), trigeminal nerve (Tg; blue), olfactory bulb (OB; orange), and brain. Tissue of Tg was collected from region shown by blue arrow. Graphs show the amount of *S. agalactiae* isolated from tissues 4 h and 24 h after intranasal inoculation for the four groups, (1) PBS → PBS (data not shown as no bacteria was isolated, **Supplementary Data Table 1**), (2) methimazole → PBS (data not shown as no bacteria was isolated, **Supplementary Data Table 1**), (3) PBS → *S. agalactiae* and (4) methimazole → *S. agalactiae*. **(B)** olfactory mucosa, **(C)** olfactory bulb, **(D)** trigeminal nerve and **(E)** brain. Data are pooled results from two independent biological experiments and are shown as median and interquartile range (4 h timepoint = 9 mice per group, 24 h timepoint = 12 mice). The *S. agalactiae* load from the different groups were compared using two-way ANOVA with significance determined using Tukey’s multiple comparison test, *p ≤ 0.05, ***p ≤ 0. 001. For mice with zero bacterial counts, these were assigned a value of 1 in order to plot on log_10_ y-axis.

Cranial nerves extending between the nasal cavity and the brain constitute a route by which certain microbes can invade the central nervous system (CNS). These nerves are the olfactory nerve and the intranasal branches of the trigeminal nerve. The cell bodies of sensory (primary) olfactory neurons are localized in the olfactory neuroepithelium of the nasal cavity. Their dendrites extend into the nasal epithelium, where odorant molecules are detected, and their axons reach all the way into the olfactory bulb in the brain, where they connect with second order neurons. The axons of sensory olfactory neurons together constitute the highly fasciculated olfactory nerve. The olfactory nerve is thus a direct connection between the periphery and the brain (Graziadei and Monti Graziadei, 1985). The anatomy of the trigeminal nerve is distinctly different; the cell bodies of its sensory neurons are found in the trigeminal ganglia, and there are only trigeminal axons near the nasal epithelium. The distal ends of these axons, however, are localized very close to the apical surface of the nasal epithelium (Schaefer et al., 2002). Thus, both the olfactory and the trigeminal nerves constitute potential invasion paths for infectious agents (Dando et al., 2014) (**Figure 1A**).

The nasal epithelium constitutes a physical barrier against microbial infection of underlying nerves, and exhibits powerful innate and adaptive immune system components, supported by the nasal-associated lymphoid tissue (NALT) (Jochems et al., 2019). Should microbes penetrate this layer, they encounter the glia of the olfactory and trigeminal nerves, olfactory ensheathing cells (OECs) and trigeminal Schwann cells (TgSCs), respectively. OECs and TgSCs have innate immune functions and are considered to be the key phagocytes in these nerves (Panni et al., 2013). The astrocytes of the glia limitans layer (the physical and immunological barrier between the peripheral nervous system and the CNS), where the nerves connect with the olfactory bulb/brainstem, are also innate immune cells and constitute a “third layer of defence” against invasion of the CNS by infectious agents (Nazareth et al., 2019). Thus, the cranial nerves and the CNS are well protected from microbial invasion, and only a small number of pathogens are thought to infect the CNS *via* these paths. Of pathogens that can use these paths, the ability to escape glial phagocytosis, and instead infect and survive inside glia, has been suggested to be a key mechanism for bacterial infection of the CNS *via* cranial nerves (Macedo-Ramos et al., 2011; Dando et al., 2014; Walkden et al., 2020).

Injuries to the nasal epithelium, for example caused by allergies, localized viral infections and mechanical trauma, are relatively common (Upadhyay and Holbrook, 2004) and may expose the underlying cranial nerves to infection. Experimental injuries to the nasal epithelium of mice have been shown to increase the risk of bacterial invasion of the olfactory nerve and bulb (Walkden et al., 2020). Premature infants are frequently fed with nasogastric tubes or intubated with nasal continuous positive airway pressure (nCPAP), which are associated with nasal injuries (Imbulana et al., 2018). Nasogastric feeding tubes are frequently contaminated with potentially pathogenic bacteria at the prong-mucosal interface (Petersen et al., 2016); thus, preterm infants may be at risk for contracting CNS infection *via* the nose-to-brain path.

*Streptococcus agalactiae* (group B streptococcus, GBS) is the leading cause of neonatal meningitis, and sometimes meningoencephalitis, affecting 0.5-3 infants per 1000 live births. *S. agalactiae* infection has high mortality and leads to permanent neurological deficits in approximately half of surviving infants (Chin and Fitzhardinge, 1985; Horváth-Puhó et al., 2021). Transmission is typically vertical (from the genouritary tract of the mother to the naso- and oropharynx of the child during delivery) (Yadeta et al., 2018), but horizontal transmission can also occur (Morinis et al., 2011). *S. agalactiae* infection can cause two clinical syndromes: early-onset disease (EOD) in the first week of life, or late-onset disease (LOD), occurring in infants aged one week to three months. Infants born prematurely also have three- to 30-fold increased risk of developing both EOD and LOD than full-term infants (Melin, 2011; Nanduri et al., 2019; Mynarek et al., 2021). It has been suggested that contaminated feeding tubes may be a source of transmission for *S. agalactiae* (Le Doare and Kampmann, 2014; Jauneikaite et al., 2018). After delivery of breast milk *via* nasogastric feeding tubes, preterm infants have been shown to develop LOD, sometimes with meningitis (Olver et al., 2000).

Although less commonly, *S. agalactiae* can also cause meningitis in adults, with increasing incidence in recent years. Nosocomial *S. agalactiae* disease in adults can arise from new acquisitions or from pre-existing colonization of skin and mucosal tissues, and droplet transmission has been described. *S. agalactiae* meningitis particularly affects people with severe underlying conditions, can be fatal or result in serious sequelae (Gupta et al., 2018). Gastric tube feeding has also led to increased amounts of *S. agalactiae* in the oral flora of elderly patients, suggesting tube contamination (Takeshita et al., 2011).

*S. agalactiae* can cross the blood-brain barrier (Maisey et al., 2008), but the mechanisms for CNS invasion have not been fully elucidated and other paths may also be involved (Dando et al., 2014). *S. agalactiae* can colonize the nasal epithelium in infants (Foster-Nyarko et al., 2016) and, whilst specific data regarding colonization of the nasal mucosa in adults is lacking, *S. agalactiae* can be cultured from the oropharynx and/or respiratory tract of ~20-25% of adults (Roloff et al., 2018). Because other commensal bacteria in the nasopharynx that cause CNS disease can reach the brain *via* the nose-to-brain cranial nerve path, we hypothesized that this path constitutes a potential mechanism by which *S. agalactiae* can infect the CNS. *S. agalactiae* also exhibits capacity for intracellular survival in a variety of cell types, including macrophages, neutrophils and microvascular endothelial cells (Cumley et al., 2012); thus, perhaps *S. agalactiae* may also survive in glia.

In the current study, we investigated whether *S. agalactiae* sequence type 17 (ST17); serotype III, which is epidemiologically the most relevant in neonatal (Edmond et al., 2012) and adult (Paveenkittiporn et al., 2020; Vuillemin et al., 2020) Group B streptococcal meningitis, can infect the brain *via* the olfactory and/or trigeminal nerves in mice. Because epithelial injury is associated with increased infection of the olfactory nerve, we also determined the effects of prior experimental injury to the nasal epithelium on *S. agalactiae* infection *via* this path. As the ability to infect glia is thought to be important for bacterial invasion of both cranial nerves and the brain, we also determined how the key glial types in the olfactory/trigeminal nerves and glia limitans layer responded to *S. agalactiae*.

## Methods

### Bacterial Strains and Culture Conditions

Wild-type (WT) *S. agalactiae* strain 874391, which is a serotype III isolate and a member of the hypervirulent ST17 lineage (Sullivan et al., 2017), and an isogenic Δ*cpsE* strain (Sullivan et al., 2016) deficient in capsule synthesis, were routinely grown at 37°C in Todd-Hewitt broth (Thermo Fisher Scientific) with shaking at 180 rpm for 16 h. Retrospective colony counts for cultures were performed by serial dilution in phosphate-buffered saline (PBS) and plating onto tryptone soya agar plates or selective media as described below.

### Ethics

The experimental procedures used in the study were conducted with the approval of the Griffith University Biosafety Committee (NLRD/09/15_var7) and the Griffith University Animal Ethics Committee (MSC/08/18/AEC) in accordance with guidelines of the Australian Commonwealth Office of Gene Technology Regulator and the National Health and Medical Research Council of Australia.

### Mouse Models of Infection and Epithelial Injury

*S. agalactiae* infection: 7- 10 weeks old female BALB/c mice (Animal Resource Centre) under anaesthesia with isofluorane (1.5-2%) were intranasally inoculated with 10 μL containing 1x10^6^ colony-forming units (CFUs) of WT *S. agalactiae* suspended in PBS, or vehicle (PBS only) as described previously for other bacteria (Nazareth et al., 2021). Mice were sacrificed 4 h and 24 h post inoculation by rising CO_2_ asphyxiation and tissues (olfactory mucosa, olfactory bulb, brain, trigeminal nerve (from within the cranial cavity), lungs, liver and blood) were collected. Tissues were homogenized using a TissueLyzer (QIAGEN) using 5 mm tungsten carbide beads and homogenates were suspended in 1 mL PBS for colony count assays by plating serial dilutions on CHROMID Strepto B agar (Biomerieux Cat number: 43461) or fixed for use in immunohistochemistry assays according to our established protocols (Nazareth et al., 2021).

For *S. agalactiae* infection post methimazole-induced epithelial injury, 7-10 weeks old female BALB/c mice (Animal Resource Centre) under anaesthesia with isofluorane (1.5-2%) were injected with methimazole (Sigma-Aldrich, 50 mg/kg, 10 mg/ml in PBS) or vehicle (PBS) using intraperitoneal injection according to our published protocol (Walkden et al., 2020). The administration of methimazole causes tissue-specific death of olfactory neurons and supporting cells secondary to degeneration of the olfactory epithelium in rodents which constitutes a more robust model of olfactory nerve injury than that caused by other chemicals (Herbert et al., 2012). Three days later when neuroepithelial damage peaks, animals were intranasally inoculated with WT *S. agalactiae* or vehicle as described above. Mice were sacrificed 4 h and 24 h post inoculation and used for colony count or immunohistochemistry assays as described above.

### Immunohistochemistry

Following sacrifice, heads of mice were fixed in 4% paraformaldehyde (PFA) overnight at 4°C, then decalcified using 20% ethylenediaminetetraacetic acid (EDTA) for 4 weeks and embedded in optimal cutting temperature (OCT) medium (ProSciTech) for sectioning. Tissue sections (50 µm cryostat sections in sagittal planes) were blocked with 2% bovine serum albumin (BSA) in PBS with 0.3% Triton-X100 solution for 60 min at room temperature. Sections were then incubated with rabbit anti-*S. agalactiae* (primary antibody; Abcam, Ab53584, 1:400), diluted in PBS-Triton-X100 overnight at 4°C, then washed and incubated with secondary antibody donkey anti-rabbit IgG (highly cross-adsorbed and conjugated to Alexa Fluor 488; 1:500; Thermo Fisher Scientific, A21206) at room temperature for 1 h, washed and stained with 4’,6-diamidino-2-phenylindole (DAPI, 1:5000 Thermo Fisher Scientific, D1306).

### Culture of Primary Glia

Primary glia cultures were obtained from S100β-DsRed transgenic mice (Windus et al., 2007), in which all glia express the bright red protein DsRed, as previously described (Nazareth et al., 2020). Briefly, the olfactory bulb and trigeminal ganglia were dissected out for preparations of OECs and TgSCs, respectively, from postnatal day 7 pups. Tissue explants were plated into 24-well plates, with wells pre-coated with Matrigel basement membrane matrix (Corning Matrigel Basement Membrane Matrix, FAL354234). The explants were maintained in glial medium, constituting of Dulbecco’s Modifed Eagle Medium (DMEM) (Gibco) with 10% fetal bovine serum (FBS), 50 μg/mL gentamycin (Gibco), 200 μM L-glutamine (Gibco) and G5 supplement diluted as per manufacturer’s instructions (Gibco). This established method yields 80% DsRed-positive cells which are also positive for glial markers (the p75 neurotrophin receptor, s100) (Nazareth et al., 2020).

Primary astrocytes with approximately 70-80% purity were obtained from the cortices of postnatal day 3 pups following a previous published protocol (Schildge et al., 2013). The cortices were dissected out and cut into small pieces in glial medium. The cortical cell suspension was then plated in a poly-D-lysine hydrobromide (Sigma-Aldrich P6407)-coated T75 flask. Following 7 to 8 days (90% confluency), astrocytes were separated from microglia and oligodendrocyte precursor cells by shaking the flask on an orbital shaker as previously described (Schildge et al., 2013). All the glia were cultured to 80% confluency after which they were trypsinized (using Gibco TrypLE Express, 1X) and used for experiments.

### *In Vitro* Infection of Primary Glia

To analyse the interactions of WT *S. agalactiae* and the capsule-deficient Δ*cpsE* mutant with glia, we exposed cultured primary OECs, TgSCs and astrocytes to the bacteria (n = 3 biological and 3 technical replicates for each condition/cell type). Methods for the routine culture and cell infection of cells were performed as previously described (Nazareth et al., 2021) with the following modifications: glia were seeded in 96-well plates at 6000 cells per well and incubated at 37°C in 5% CO_2_ until approximately 80% confluence was achieved. Glial cell monolayers were then infected with *S. agalactiae* at a multiplicity of infection (MOI) of 100 bacteria per cell (or, for control, medium alone was added to the cultures) for 1 h. The plate was then incubated at 37°C in 5% CO_2_. Bacteria were initially allowed to attach to glia for 1 h, before the infecting inoculum was aspirated and unattached bacteria was removed by washing the well three times with PBS. Then, adherent bacteria were enumerated by colony counts on Todd-Hewitt agar.

To determine the concentration of intracellular bacteria, antibiotic protection assays were utilised (Dando et al., 2016). Adherent extracellular bacteria were removed by the addition of glial medium containing antibiotics (penicillin 250 U/mL and streptomycin 250 U/mL with gentamycin 50 µg/mL, Gibco) and the number of intracellular bacteria was determined at 2 h and 24 h post infection.

### Immunocytochemistry

Glial cells were seeded onto 96-well plates at 6000 cells per well and the following day were infected with WT or Δ*cpsE S. agalactiae*, or medium alone (control). Infected monolayers at various times were fixed with 4% PFA for 10 min, rinsed with PBS, followed by blocking/permeabilising solution (3% BSA in PBS with 0.3% Triton X-100) for 30 min at room temperature on a shaker.

Rabbit anti-*S. agalactiae* (Abcam, Ab53584; 1:400) or goat anti-glial fibrillary acidic protein (GFAP) (Abcam, ab53554; 1:400) was added to the fixed cells which were kept at 4°C overnight on a rocking shaker. The following day, plates were washed with PBS and secondary antibodies were added. These were donkey anti-rabbit IgG (Alexa Fluor 488; 1:500; Thermo Fisher Scientific, A21206), or donkey anti-goat IgG (Alexa Fluor 647; 1:500; Abcam, ab150135). Cell nuclei were stained with 4’,6-diamidino-2-phenylindole (DAPI,1:5000 Thermo Fisher Scientific, D1306). To assess cytotoxicity of glial cells after infection, live cell nuclei were stained with Hoechst 33342 (1:1000, Thermo Fisher Scientific, H1399) and dead cells were stained with DRAQ7 Dye (1:500; Thermo Fisher Scientific, D15106).

For glial mediated phagocytosis of bacteria in lysosomes, rat anti-lysosomal membrane protein 2 marker antibody (LAMP2) [GL2A7] (1:800, Abcam, ab13524) and chicken anti-glial fibrillary acidic protein (GFAP) (Abcam, ab4674; 1:800) was used with the rabbit anti-*S. agalactiae* (Abcam, Ab53584; 1:400) at 4°C overnight on a rocking shaker. Combinations of appropriate secondary antibodies were used: goat anti-chicken IgY H&L 647 (1:1000, Abcam, ab150171) plus goat anti-rat IgG H&L (Alexa Fluor 488) (1:500, Abcam, ab150157) and goat anti-rabbit IgG H&L (Alexa Fluor 594) preadsorbed (1:500, Abcam, ab150084); with Hoechst 33342 (1:5000, Thermo Fisher Scientific, H1399) to stain nuclei.

### Imaging

All high magnification images were collected using an Olympus FV3000 confocal microscope and panels show confocal images of maximum projection of z-stacks. Images were colour-balanced using Adobe Photoshop CS5 (Adobe Systems Incorporated) with the figures being compiled in Adobe Illustrator CS5 (Adobe Systems Incorporated). 3D reconstruction of z-stacks were generated and videos were compiled using Imaris 9.5.1 software.

### Cytokine and Chemokine Assays

The levels of cytokines were determined in the cell culture supernatants at 24 h post inoculation with Bio-Plex Pro Mouse Cytokine 23-plex assays (Bio-Rad, Gladesville, NSW, Australia). Cell culture supernatants were collected and centrifuged at 1,000 × *g* for 15 min at 4°C. Samples were then stored at −80°C until analysis. Thawed samples were then assayed according to the manufacturer’s instructions. The plates were read on a BioPlex 200 Luminex bead array reader (Bio-Rad) and data were acquired with Bio-Plex Manager Software (version 5.0; Bio-Rad).

### Data Analysis

Statistical testing and graphical analysis were conducted using GraphPad Prism version 8.0. Colony count data from organ lysates and from cell lysates were examined by Kruskal-Wallis Test followed by Dunn’s multiple comparison post-tests. Cytokine and chemokine expression data were compared using two-way analysis of variance (ANOVA) followed by Tukey’s multiple comparison *post-hoc* tests.

## Results

### *S. agalactiae* Can Invade the CNS *via* the Olfactory Nerve

To determine whether *S. agalactiae* could invade the CNS *via* the olfactory nerve and/or the intranasal branches of the trigeminal nerve, we intranasally inoculated mice with *S. agalactiae* (10^6^ bacteria in 10 μL) and determined whether live *S. agalactiae* bacteria could be isolated from mucosal, nerve and CNS tissues. Control inoculations contained PBS only (vehicle). At 4 h and 24 h post inoculation, the mice were sacrificed and homogenates of the olfactory mucosa, olfactory bulb, trigeminal nerve, brain (beyond the olfactory bulb; **Figure 1A**), lungs, liver and blood (**Supplementary Data, Table 1**) were analysed for *S. agalactiae* load. For the control group (PBS → PBS), no bacteria were isolated from any of the tissues (olfactory mucosa, olfactory bulb, trigeminal nerve, brain, lungs, liver and blood; **Supplementary Data, Table 1** and **Figures 1B–E**). For the *S. agalactiae* infected group at 4 h, median tissue load was 4 log_10_ CFU/0.1g tissue (interquartile range (IQR) 3.3-4.4) in olfactory mucosa and no bacteria were present in all the other organs tested (**Figures 1B–E**). For the *S. agalactiae* infected group at 24 h post infection, median tissue loads were 3.6 log_10_ CFU/0.1g tissue (IQR 0.8-4.3) in olfactory mucosa, 3 log_10_ CFU/0.1g tissue (IQR 0-3.8) in the olfactory bulb, and 1.6 log_10_ CFU/0.1g tissue (IQR 0.2-2.2) in the brain (**Figures 1B–E**). Interestingly, however, no *S. agalactiae* bacteria were isolated from the trigeminal nerve, lungs, liver, and blood 4 h and 24 h post infection (**Figure 1D**; **Supplementary Data Table 1**).

### Injury to the Nasal Epithelium Exacerbates Infection *via* the Olfactory Nerve Route and Results in Infection of the Trigeminal Nerve

As injury to the olfactory epithelium has been shown to increase the amount of bacteria infecting the olfactory nerve/bulb (Walkden et al., 2020), in parallel assays to those described above we pre-injured the olfactory neuroepithelium with our well-established method using methimazole (Walkden et al., 2020), which causes death of primary olfactory neurons and damage to the olfactory epithelium. Three days later, we inoculated the mice intranasally with *S. agalactiae*. At this time, death of olfactory neurons is at its peak (Nazareth et al., 2015) and methimazole has been cleared, limiting potential unknown side-effects of methimazole (Xie et al., 2011). Epithelial injury significantly increased the bacterial load isolated from the olfactory mucosa, and brain (**Figures 1B, C, E**) at 4 h and 24 h, compared to parallel infection without injury. This also resulted in recovery of *S. agalactiae* from the trigeminal nerve with median 3.4 log_10_ CFU/0.1g tissue (IQR 2.9-3.7) at 4 h and median 3.4 log_10_ CFU/0.1g tissue (IQR 0.7-4) at 24 h post infection (**Figure 1D**). Also, no *S. agalactiae* bacteria were isolated from the lungs, liver, and blood 4 h and 24 h post infection. Thus, *S. agalactiae* can infect the CNS *via* the olfactory nerve, and epithelial injury resulted in significantly elevated numbers of invading bacteria in the brain and olfactory bulb. Epithelial injury also exposes the trigeminal nerve for *S. agalactiae* infection.

**Figure 2:**
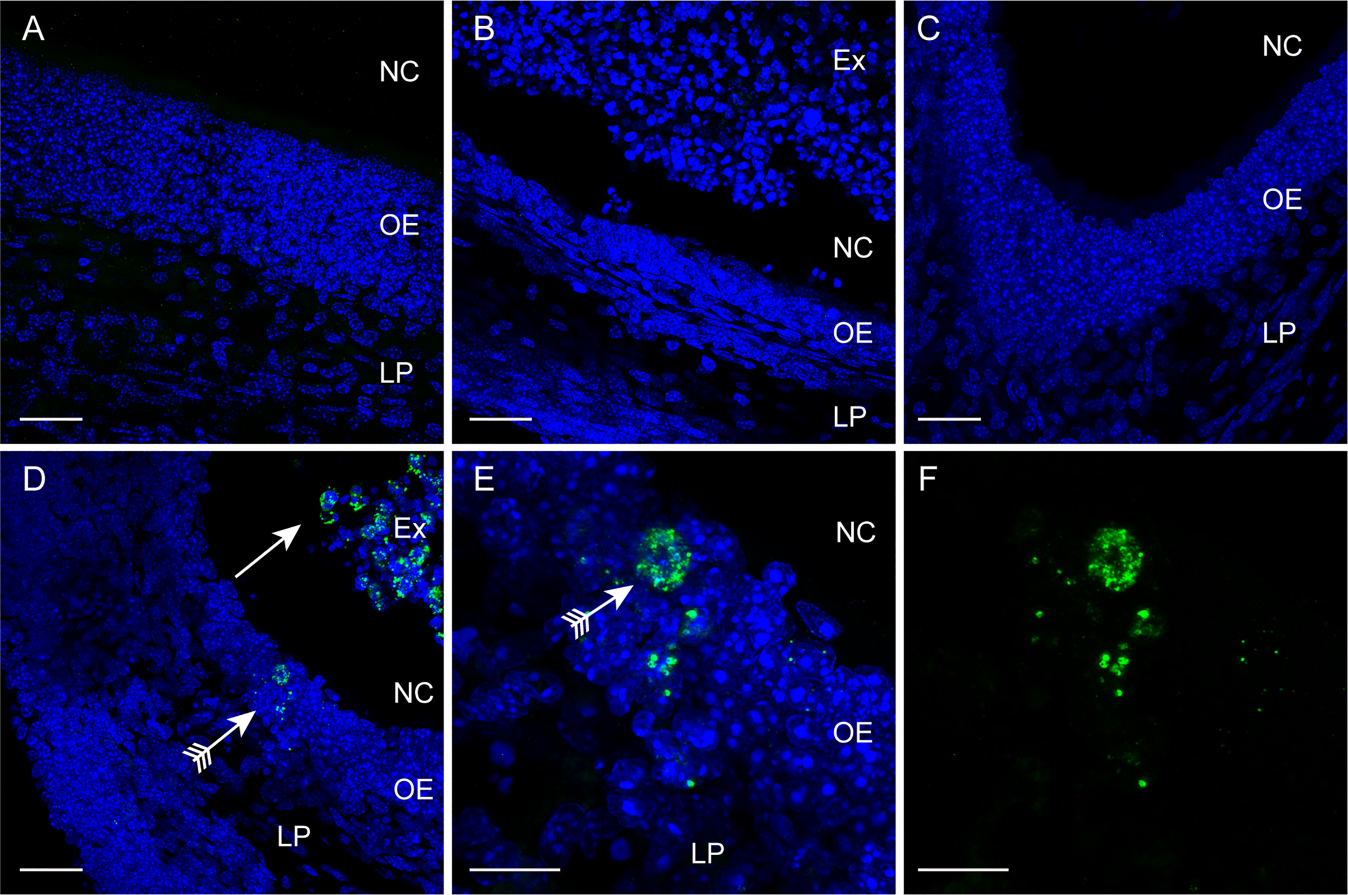
Immunolabelling for *S. agalactiae* in the olfactory mucosa. Panels show confocal microscopy images of the olfactory mucosa with immunolabelling for *S. agalactiae* (green), and nuclei stained with DAPI (blue) 24 h post intranasal inoculation with the bacteria or vehicle. **(A)** Control, vehicle-only (PBS → PBS) treatment. The olfactory epithelium (OE) and lamina propria (LP) constitute the olfactory mucosa. NC: nasal cavity. **(B)** Methimazole treatment only (methimazole → PBS). The OE has been degraded and is sloughing off as an exudate (Ex) into the NC. **(C)** Intranasal inoculation with *S. agalactiae* (PBS → *S. agalactiae*). No *S. agalactiae* bacteria were detected within the OE. **(D)** Methimazole treatment followed by intranasal *S. agalactiae* inoculation (methimazole → *S. agalactiae*). *S. agalactiae* bacteria were detected in the Ex (arrow) and in the OE (arrow with tail). **(E, F)** Higher magnification view of the OE shown in **(D)**; **(F)** *S. agalactiae* immunolabelling alone. Scale bar: 30 μm in **(A–D)**; 10 μm in **(E, F)**.

To further examine the dynamics of *S. agalactiae* invasion *via* the CNS, we immunolabelled tissue sections from the olfactory epithelium, bulb and brain for *S. agalactiae*. In the control uninfected animals (PBS → PBS), the olfactory epithelium was an intact layer of dense cells (**Figure 2A**). After methimazole treatment alone (methimazole → PBS), the epithelial layer sloughed off into the nasal cavity (**Figure 2B**). When *S. agalactiae* bacteria were intranasally inoculated after methimazole injury (methimazole → *S. agalactiae*), bacteria were readily detected within the remaining tissue of the olfactory epithelium as well as within the exudate in the nasal cavity (**Figures 2D–F**). We could not detect *S. agalactiae* from the olfactory mucosa of uninjured mice (**Figure 2C**), but, consistent with a ~100-fold reduction in the number of culturable bacteria from the uninjured vs the injured group (3.6 vs 5.3 log_10_ CFU/0.1g tissue, respectively), this is likely due to sparse distribution of bacteria within the tissue. In the other tissues analysed, we could not clearly distinguish *S. agalactiae* cocci from background immunolabelling and thus, we only show immunolabelling data for the olfactory epithelium.

### *S. agalactiae* Can Invade and Survive Inside Glia

**Figure 3:**
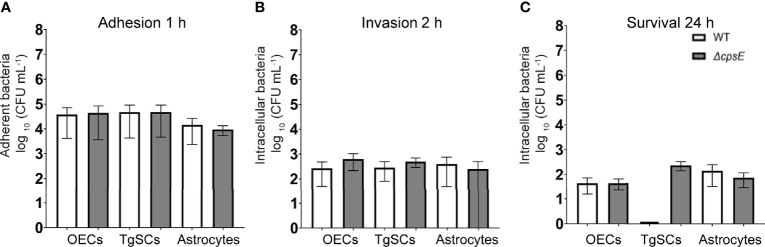
Comparison of adhesion/invasion of glia by *S. agalactiae*. **(A)** Cell monolayers were inoculated with WT or Δ*cpsE* bacteria for 1 h (MOI 100), followed by colony counts to determine the number of adherent bacteria. **(B, C)** After 1 h, penicillin, streptomycin and gentamicin were added to the medium to kill extracellular bacteria, and at 2 h **(B)** or 24 h **(C)** cells were lysed to recover intracellular bacteria and colony counts were performed. Data shows mean ± SEM of three independent experiments with n = 3 technical replicates. Data were compared between glial types using two-way ANOVA with Tukey’s multiple comparison test; no significant differences were detected. CFU/mL values of zero were assigned a value of 1 to enable display on log10 axes (in graph **(C)**, TgSCs WT value is zero).

**Figure 4:**
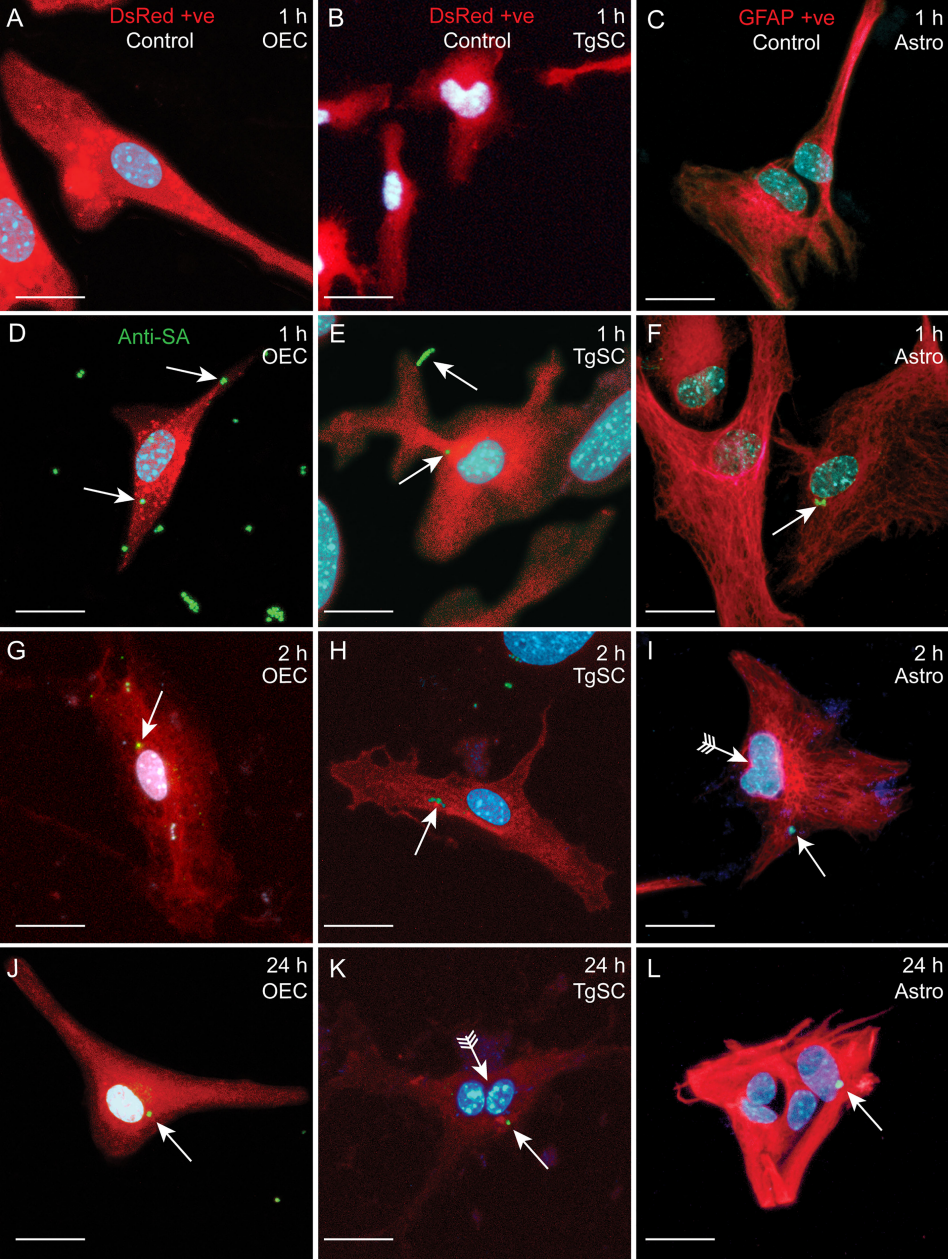
Confocal images showing attachment and internalisation of *S. agalactiae* in glia (OECs, TgSCs and astrocytes). **(A–C)** Control cells (without *S. agalactiae*). OECs and TgSCs were visualised by DsRed expression (red), whilst astrocytes were immunolabelled for GFAP (red). Nuclei are stained with DAPI (blue). **(D–L)** Representative images of OECs, TgSCs and astrocytes following inoculation with *S. agalactiae* (anti-*S. agalactiae* antibody (anti-SA); green, arrow). After 1 h of exposure to *S. agalactiae* **(D–F)**, extracellular bacteria were removed by washing and addition of antibiotic in the medium. *S. agalactiae* were detected in the glia at 2 h **(G–I)** and at 24 h post infection **(J–L)**. Some cells exhibited atypical nuclei or binucleation (arrows with tail). Scale bar: 20 μm.

Glia (OECs and TgSCs) are the key phagocytes in the olfactory and trigeminal nerves, respectively, whilst astrocytes (CNS glia) in the glia limitans layer between the nerves and the CNS constitute another layer of defence against pathogens. As the capability to infect these glia is thought to constitute a key mechanism for CNS invasion, we therefore determined the susceptibility of these glia to *S. agalactiae* infection. Using antibiotic protection assays, we compared the adhesion and invasion capacity of *S. agalactiae* between primary OECs, TgSCs and astrocytes. To investigate bacterial adhesion to the cells, we exposed the cells to *S. agalactiae* at a multiplicity of infection of 100 bacteria per cell (MOI 100) for 1 h and determined the number of bacteria that had attached to the cells. To determine whether *S. agalactiae* could invade and survive within OECs, TgSCs and astrocytes, an antibiotic cocktail was added to the medium to kill extracellular bacteria. Cells were then incubated for a further 2 h or 24 h and the number of intracellular bacteria was determined for the different cell types by lysing monolayers at these timepoints. We found that *S. agalactiae* adhered to the cells within 1 h (**Figure 3A**), and bacteria could be recovered from all glia at 2 h post antibiotic addition, representing invasive *S. agalactiae* (**Figure 3B**). The frequency of invasion (% of adhered bacteria recovered at 2 h post antibiotics) was similar for the three glial types (**Supplementary Figures 1A, B**). After 24 h, however, bacteria were only recovered from OECs and astrocytes, and not in TgSCs (**Figure 3C** and **Supplementary Figure 1C**) suggesting that *S. agalactiae* was eliminated from TgSCs at 24h post inoculation. To assess the cytotoxicity of *S. agalactiae* on glial cells, percentage of dead cell count and total cell count was performed at 1 h and 24 h post infection. No significant cytotoxicity was observed in glial cells (OECs, TgSCs and astrocytes) post infection (**Supplementary Figure 2**). Thus, the trends observed in the results are due to glial responses to bacteria and not due to cell death occurring post infection. To resolve the interactions of *S. agalactiae* with OECs, TgSCs and astrocytes at the cellular level, we fixed and imaged infected cells at 1 h, 2 h and 24 h time-points. The glia was isolated from S100β-DsRed mice, in which the S100 promoter drives the expression of the fluorescent protein DsRed in glia, allowing easy visualisation of the cells. The expression of DsRed is lower in astrocytes than in the other glia; therefore, the identity of these cells was verified using immunolabelling for glial fibrillary associated protein (GFAP), a well-established astrocyte marker (Schildge et al., 2013) (**Figures 4C, F, I, L**). *S. agalactiae* immunolabelling showed that bacteria were attached to cells at 1 h (**Figures 4D–F**) and were found inside cells at 2 and 24 h post exposure (**Figures 4G–L**). Although bacterial counts were below the detection limit from TgSCs at 24 h post inoculation (**Figure 3C**), we could occasionally find *S. agalactiae* cocci in some TgSCs using imaging (**Figure 4K**). We observed that some of the cells were bi- or multinucleated, or had atypical nuclei (**Figures 4I, K, L**).

### The *S. agalactiae* Capsule Contributes to Invasion and Survival Within Trigeminal Schwann Cells

**Figure 5:**
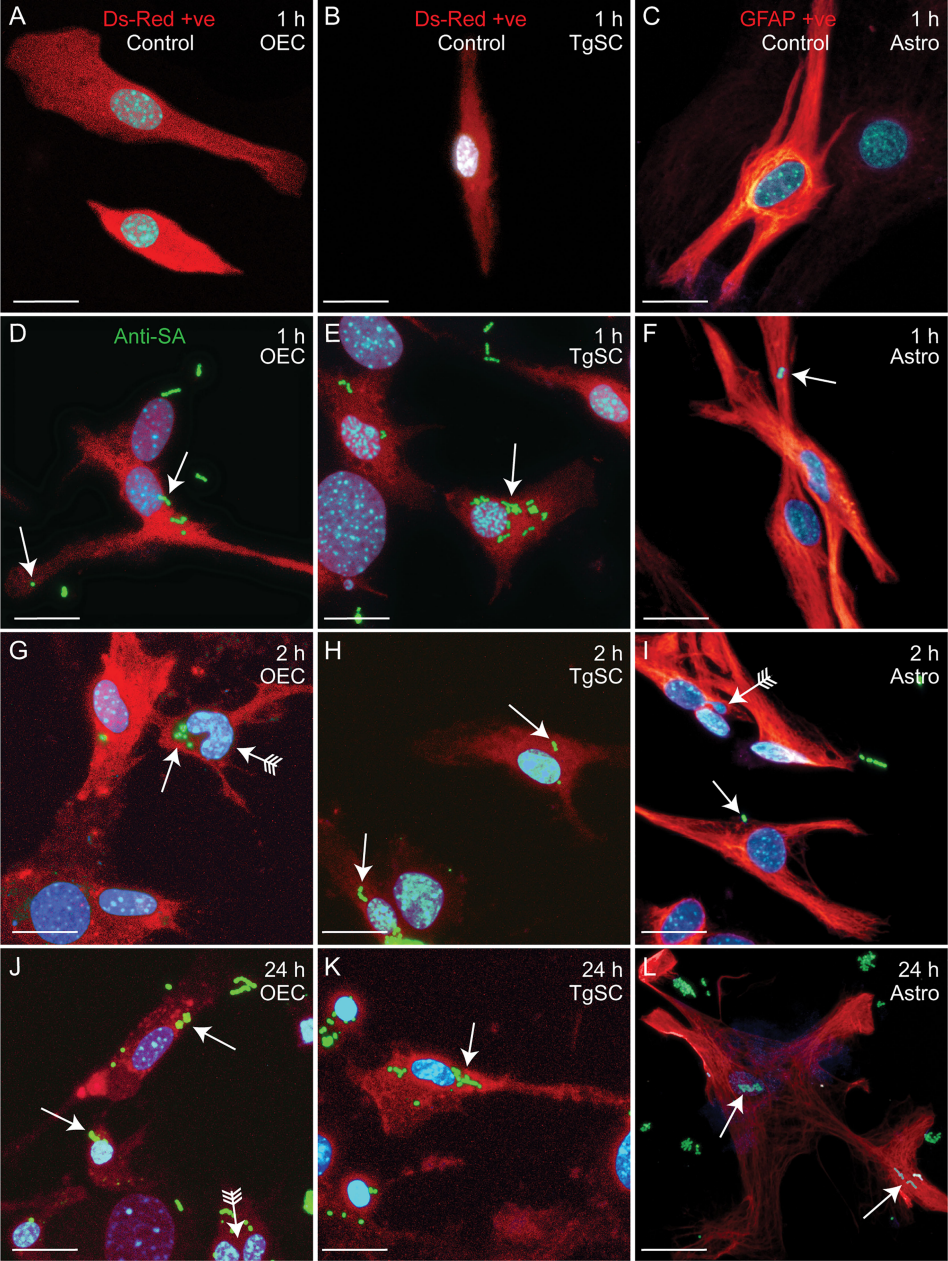
Confocal images showing attachment and internalisation of Δ*cpsE S. agalactiae* in the three types of glia. **(A–C)** Control cells (no *S. agalactiae*). Colours and immunolabelling/staining are consistent with **Figure 4** (red: glia, blue: nuclei, green: Δ*cpsE*). **(D–F)** Representative images of OECs, TgSCs and astrocytes 1 h after inoculation with Δ*cpsE* (green, arrow). **(G–L)** Δ*cpsE* were detected in glia at 2 h **(G–I)** and at 24 h post inoculation **(J–L)**. Some cells exhibited atypical nuclei or binucleation (arrows with tail). Scale bar: 20 μm.

Lack of *S. agalactiae* polysaccharide capsule may alter colonization and cellular responses to the bacteria (Alkuwaity et al., 2012; Sullivan et al., 2016). Therefore, in parallel assays, we compared glial responses to an isogenic *S. agalactiae* mutant in *cpsE* (Δ*cpsE*) that is devoid of capsule (Sullivan et al., 2016). We found that colony counts of adhesion, invasion and intracellular survival for the Δ*cpsE* strain were similar to WT for OECs, TgSCs and astrocytes (**Figures 3A–C**); while the two-way ANOVA analysis did not reveal significant differences between any of the treatment groups, for the 24 h intracellular survival in TgSCs there was a p value of 0.08 for the comparison between Δ*cpsE* strain and WT. Interestingly, with a comparison of percentage of bacteria load to initial inoculum, there was significantly more Δ*cpsE S. agalactiae* recovered from TgSCs at 24 h compared to WT (p ≤ 0.001, **Supplementary Figure 1C**). In addition, Δ*cpsE* bacteria also had significantly higher capacity for intracellular survival in TgSCs than in astrocytes (both WT and Δ*cpsE*, p≤ 0.01) and also compared to OECs (WT only, ≤ 0.01) (**Supplementary Figure 1C**). Imaging confirmed the Δ*cpsE* strain could adhere to, invade and survive inside all three glial types (**Figure 5**).

### Phagocytosis of *S. agalactiae* in Phagolysosomes in Glial Cells

**Figure 6:**
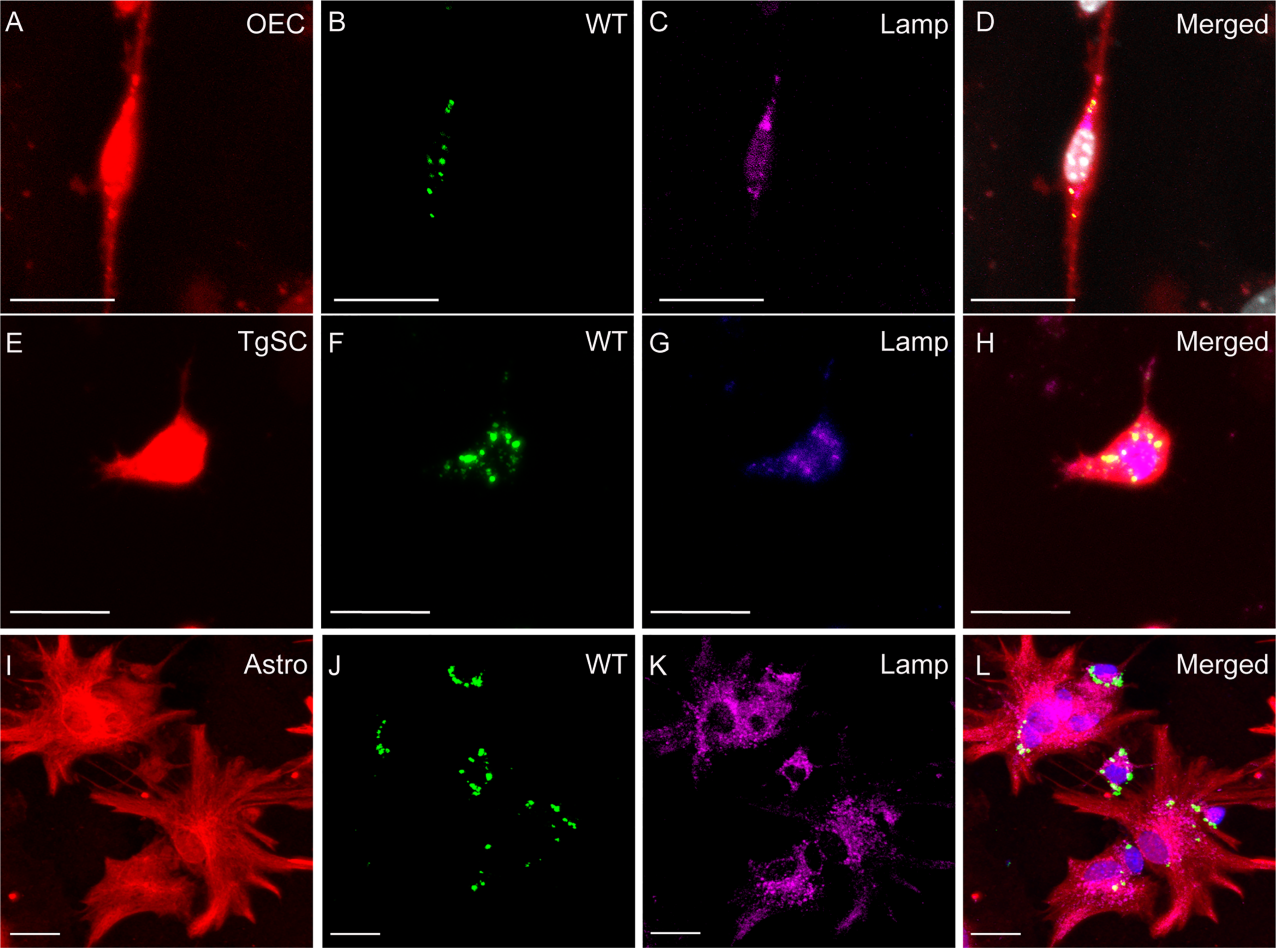
Phagocytosis of *S. agalactiae* in phagolysosomes in glial cells (OECs, TgSCs and astrocytes) 24 h post infection. Panels show primary cultures of OECs **(A–D)**, TgSCs **(E–H)** and astrocytes **(I–L)** from S100β-DsRed mice. OEC and TgSCs were visualised by DsRed expression (red), whilst astrocytes were immunolabelled for GFAP (red). Nuclei are stained with Hoechst. **(B, F, J)** shows *S. agalactiae* within cells, **(C, G, K)** LAMP-2 immunolabelling for lysosomes and **(D, H, L)** *S. agalactiae* colocalizes with LAMP-2 showing the bacteria is internalized in phagolysosomes. Scale bar: 25 μm.

**Figure 7:**
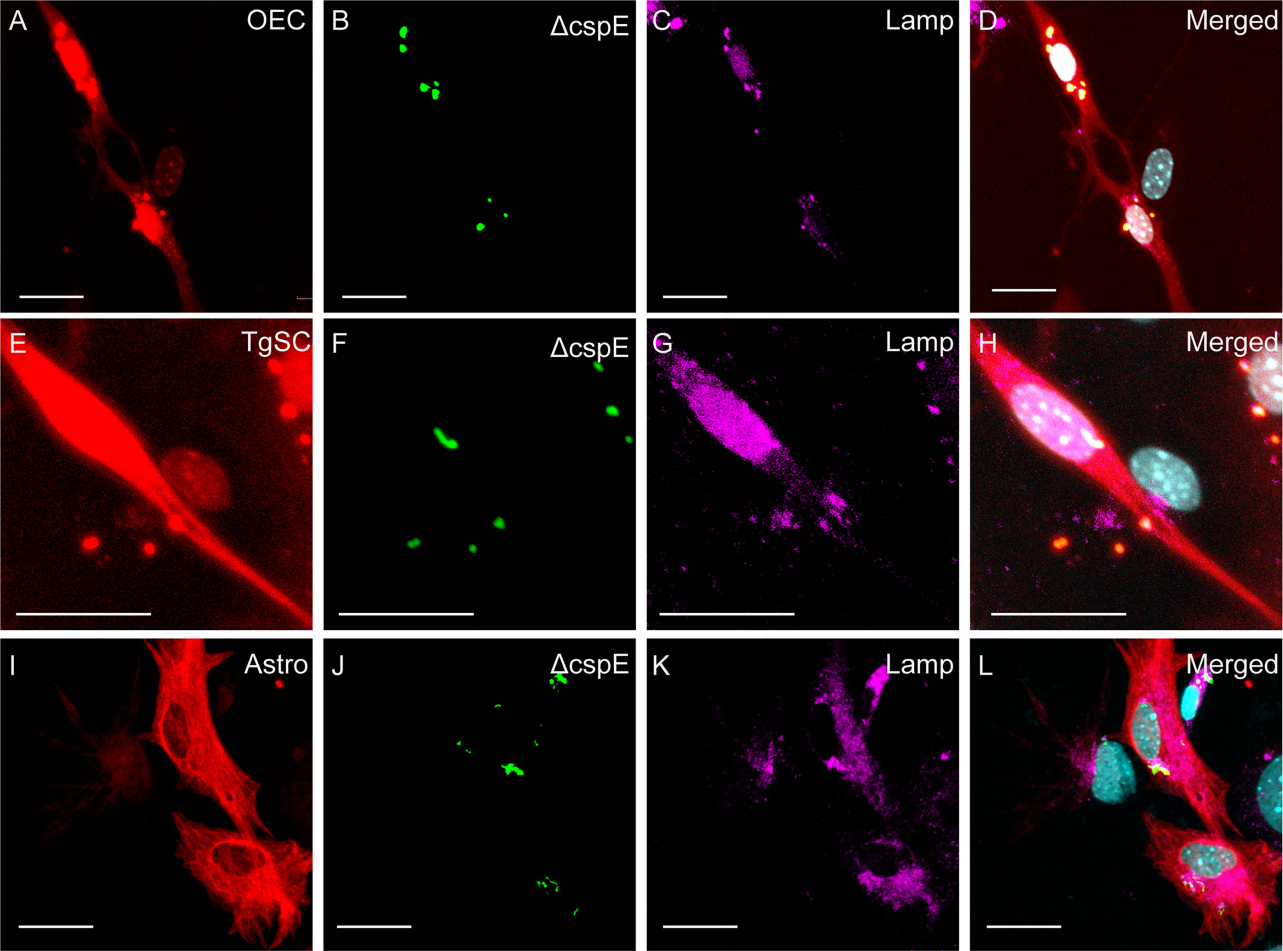
Phagocytosis of Δ*cpsE S. agalactiae* in phagolysosomes in glial cells (OECs, TgSCs and astrocytes) 24 h post infection. Panels show primary cultures of OECs **(A–D)**, TgSCs **(E–H)** and astrocytes **(I–L)** from S100β-DsRed mice. OEC and TgSCs were visualised by DsRed expression (red), whilst astrocytes were immunolabelled for GFAP (red). Nuclei are stained with Hoechst. **(B, F, J)** shows *S. agalactiae* within cells, **(C, G, K)** LAMP-2 immunolabelling for lysosomes, and **(D, H, L)** *S. agalactiae* colocalizes with LAMP-2 showing the bacteria is internalized in phagolysosomes. Scale bar: 25 μm.

To determine the fate of *S. agalactiae* after internalisation within the glia, we immunolabelled the cells at 24 h post infection for lysosome-associated membrane protein 2 (LAMP-2). LAMP-2 is the major protein component of the lysosome membrane which is required for the fusion between the late phagosome and lysosomes as well as acidification of the lysosomal lumen that helps in degradation of the internalized foreign particles such as bacteria (Eskelinen et al., 2002; Huynh et al., 2007). We observed that all glial cells (OECs, TgSCs and astrocytes) showed well-defined LAMP2-positive lysosomes around both WT and Δ*cpsE S. agalactiae* (**Figures 6**, **7**) demonstrating that the bacteria were internalized or colocalized inside lysosomes after infection. To support the above statement 3D reconstruction and videos were generated which showed co-localisation of bacteria and LAMP within the cells (**Supplementary Video 1** (OECs), **Video 2** (TgSCs) and **Video 3** (astrocytes).

### Glia Respond to *S. agalactiae* by Secreting Cytokines and Chemokines

To gain an understanding of the innate immune responses of the different glia to *S. agalactiae*, we also analysed secretion of cytokines and chemokines. As the *S. agalactiae* capsule has previously been found to alter cytokine production by other cell types (Lemire et al., 2012b), we also assessed whether the capsule affected secretion of cyto-/chemokines. We inoculated the cells with both *S. agalactiae* WT (white bars) and Δ*cpsE* strain (grey bars) and cyto/chemokine levels were analysed at 24 h post inoculation.

#### Cytokines

**Figure 8:**
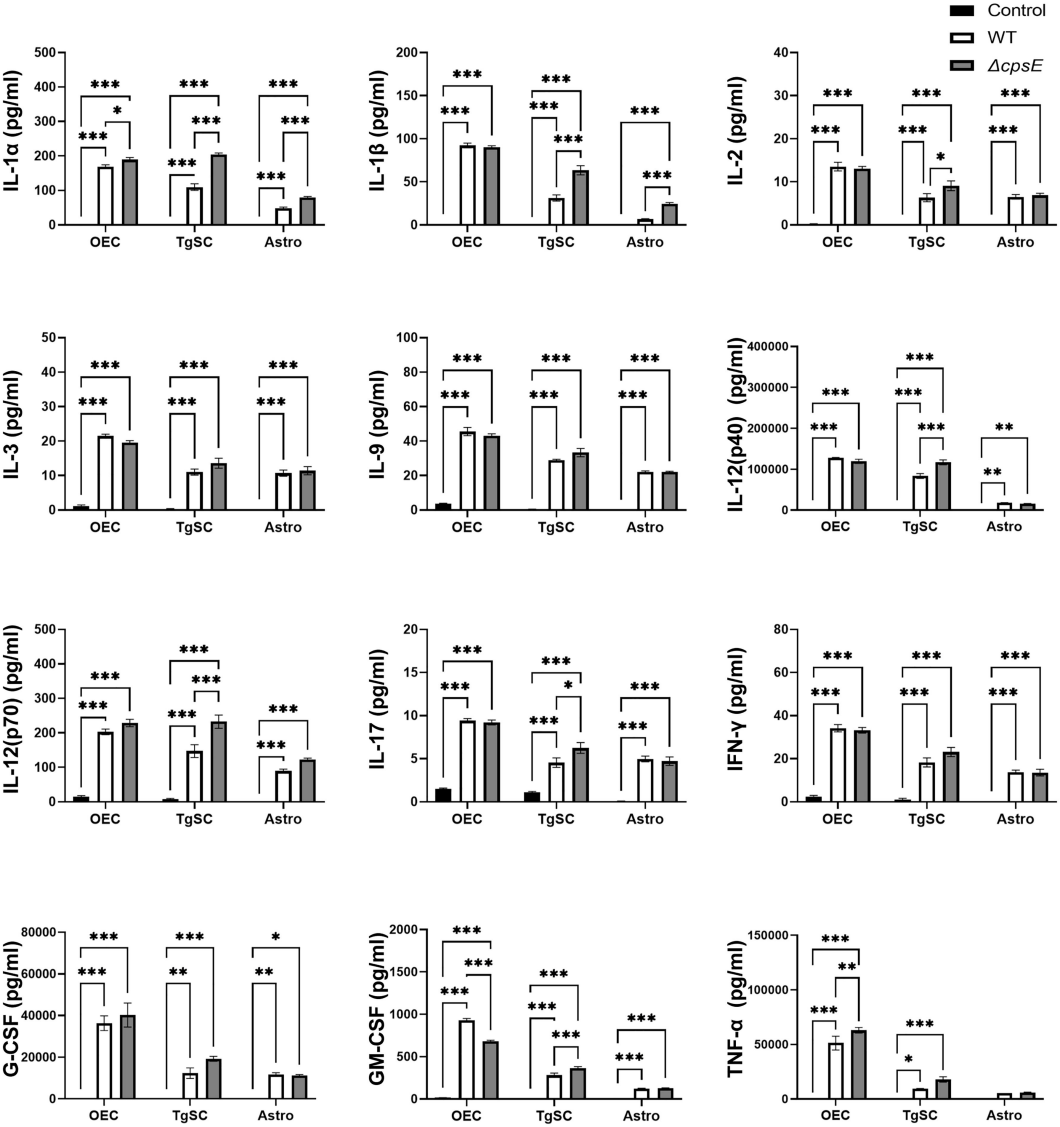
Pro-inflammatory cytokine responses in glia after inoculation with wild-type and capsule-deficient *S. agalactiae*. OECs, TgSCs and astrocytes were inoculated with either WT or Δ*cpsE* (MOI 100:1) after which the cytokine levels in the medium were analysed at 24 h post inoculation. Data show mean ± SEM, *p ≤ 0.05, **p ≤ 0.01, ***p ≤ 0.001 (two-way ANOVA with Tukey’s multiple comparison test, n = 3 technical replicates x 100,000 cells/well).

**Figure 9:**
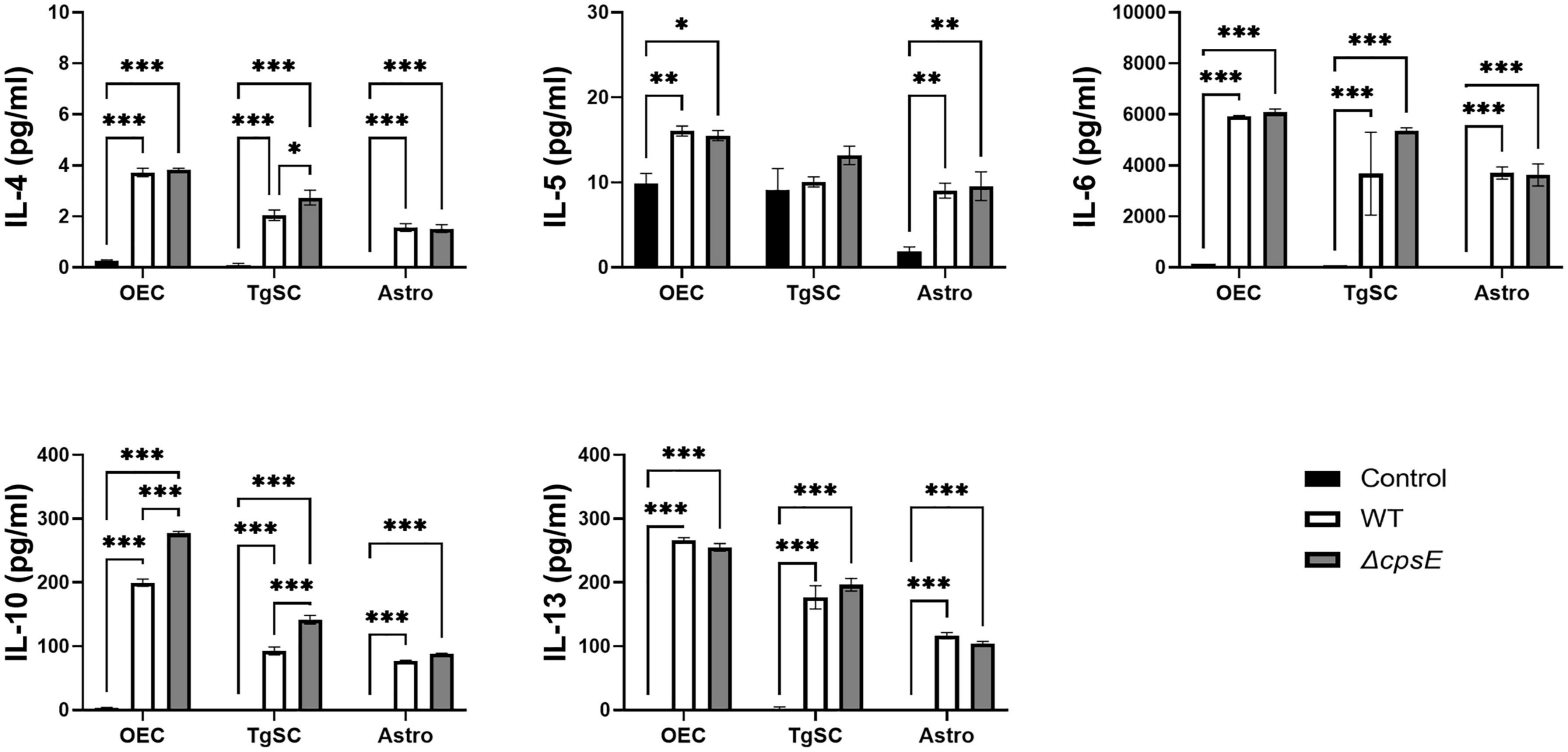
Anti-inflammatory cytokine responses in glia after inoculation with wild-type and capsule-deficient *S. agalactiae*. OECs, TgSCs and astrocytes were inoculated with either WT or Δ*cpsE* (MOI 100:1) after which the cytokine levels in the medium were analysed at 24 h post inoculation. Data show mean ± SEM, *p ≤ 0.05, **p ≤ 0.01, ***p ≤ 0.001 (two-way ANOVA with Tukey’s multiple comparison test, n = 3 technical replicates x 100,000 cells/well).

**Figure 10:**
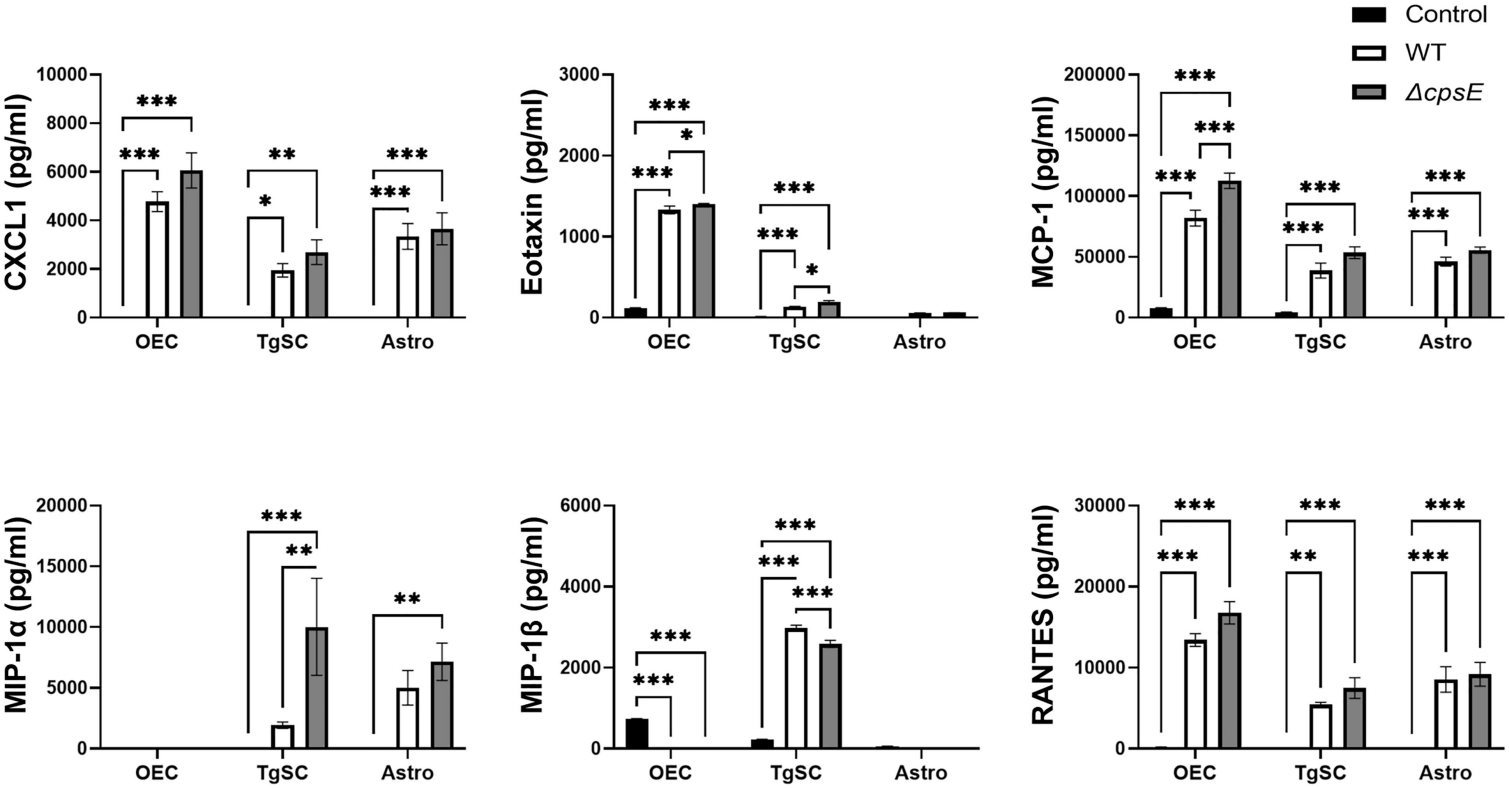
Chemokine responses in glia after inoculation with wild-type and capsule-deficient *S. agalactiae*. OECs, TgSCs and astrocytes were inoculated with either WT or Δ*cpsE* (MOI 100:1) after which the chemokine levels in the medium were analysed at 24 h post inoculation. Data show mean ± SEM, *p ≤ 0.05, **p ≤ 0.01, ***p ≤ 0.001 (two-way ANOVA with Tukey’s multiple comparison test, n = 3 technical replicates x 100,000 cells/well).

Infection of all the glial types with *S. agalactiae* WT and Δ*cpsE* strain resulted in the production of numerous pro-inflammatory cytokines (**Figure 8**), anti-inflammatory cytokines (**Figure 9**), and chemokines (**Figure 10**) at 24 h post-inoculation (all at significantly higher levels than non-infected control glia). The glial cytokine response included pro-inflammatory cytokines previously reported to be associated with *S. agalactiae* infection (Patras and Nizet, 2018), such as interleukin 1β (IL-1β), interleukin 17 (IL-17), interferon γ (IFN-γ) and tumour necrosis factor α (TNF-α). The glia also responded to both the *S. agalactiae* WT and Δ*cpsE* strain with secretion of the anti-inflammatory (regulatory) cytokines interleukin 4 (IL-4), interleukin 5 (IL-5), interleukin 6 (IL-6), interleukin 10 (IL-10) and interleukin 13 (IL-13) at 24 h post inoculation (**Figure 9**). Overall, *S. agalactiae*-infected OECs also produced higher levels of both pro- and anti-inflammatory cytokines than TgSCs and astrocytes (**Figures 8**, **9**).

The glia exhibited differential cytokine responses to the wild-type and capsule-deficient *S. agalactiae* strains. All three glia produced significantly higher levels of IL-1α (**Figure 8A**), while OECs and TgSCs produced significantly higher levels of IL-10 and only TgSCs produced significantly higher levels of IL-12p70 in response to the Δ*cpsE* strain than to the WT strain (**Figures 8**, **9**). For TgSCs in particular, the Δ*cpsE* strain was generally associated with secretion of higher levels of cytokines than the WT strain (with individual variations depending on cytokine and/or cell type; **Figures 8**, **9**). In response to Δ*cpsE* strain, TgSCs secreted significantly more IL-2, IL-12p40, IL-12p70, IL-17 than in response to WT. OECs, in contrast, secreted significantly lower levels of GM-CSF after exposure to the capsule-deficient strain than to the wild-type strain (**Figure 8K**).

#### Chemokines

The glia also responded to *S. agalactiae* with the secretion of the chemokines C-X-C motif ligand 1 (CXCL-1, also known as Gro/KC or Groα), eotaxin, monocyte chemoattractant protein 1 (MCP-1), macrophage inflammatory proteins (MIP-1α and MIP-1β, also known as CCL3 and CCL4) and regulated upon activation-normal T cell expressed and presumably secreted (RANTES) (**Figure 10**). At 24 h post inoculation, OECs secreted higher levels of CXCL-1, eotaxin, MCP-1 and RANTES than the other cell types (**Figure 10**).

Some differences in secretion of chemokines were found between cells that had been inoculated with the WT strain versus the Δ*cpsE* strain. At 24 h post inoculation, all three glial types secreted higher levels of MCP-1 in response to the capsule-deficient strain than to the wild-type strain, although only OECs had significantly higher levels. TgSCs also secreted more MIP-1α in response to the *S. agalactiae* Δ*cpsE* strain than to the wild-type strain (**Figure 10**).

## Discussion

This study demonstrates that *S. agalactiae* can rapidly (within 24 h) infect the CNS *via* the olfactory nerve in mice, and that injury to the nasal epithelium exacerbates infection *via* this route. We also found that injury, often associated with nasal infections or mechanical trauma in humans, exposes the trigeminal nerve to infection. In cultured cells, we showed (1) that *S. agalactiae* can infect and survive inside glia (primary mouse OECs, TgSCs and astrocytes), (2) resulting in glial-mediated secretion of cytokines and chemokines, and (3) that the *S. agalactiae* capsule deficiency plays a major role in cellular interactions with TgSCs and cytokine/chemokine secretion. Together, these findings show that cranial nerves extending between the nasal cavity and the brain constitute an alternative path by which *S. agalactiae* can infect the CNS, which may explain why meningitis can occur in patients despite negative blood cultures (Garges et al., 2006). The outcomes also suggest that intracellular survival in glia constitutes a mechanism behind the capacity for invading the brain *via* these routes.

The olfactory nerve has long been hypothesized to be a potential invasion route for pathogens causing bacterial meningitis (Filippidis and Fountas, 2009). Rapid infection of the meninges/CNS *via* this route has been demonstrated for *Neisseria meningitidis* (Sjolinder and Jonsson, 2010) and *Streptococcus pneumoniae* (van Ginkel et al., 2003), even in the absence of blood infection. *Chlamydia pneumoniae*, which is predominantly a respiratory pathogen, is also considered to infect the CNS *via* this path (Balin et al., 2018) and *Treponema* species (Riviere et al., 2002) appear to primarily reach the CNS *via* the trigeminal nerve. *Chlamydia muridarum* (Nazareth et al., 2021) (*Chlamydia* commonly used to model rodent chlamydia lung infection), *Burkholderia pseudomallei* (St John et al., 2014; Dando et al., 2016) and *Listeria monocytogenes* have also been shown to infect the CNS *via* both the olfactory and the trigeminal nerve routes in mice, with circumstantial evidence of trigeminal nerve/brainstem involvement in humans for the latter two (Drevets and Bronze, 2008; Pagelow et al., 2018). Certain viruses such as herpes simplex type 1 (HSV1) (Shivkumar et al., 2013; Doll et al., 2019) and SARS-CoV2 (Brouwer et al., 2020) are also considered capable of invading the brain *via* the olfactory and/or trigeminal nerve routes. The majority of these infectious agents can invade the CNS *via* the nerves rapidly (within 24-48 h) (Dando et al., 2014).

Infection of the olfactory and/or trigeminal nerve by *B. pseudomallei* (St John et al., 2014) and *N. meningitidis* (Sjolinder and Jonsson, 2010) are associated with patchy injury to the nasal epithelium. In the current study, we did not observe epithelial damage when mice were infected with *S. agalactiae* alone; despite this, the bacteria were able to enter the olfactory nerve. This is in alignment with previous studies of olfactory nerve infection by *S. pneumoniae* (van Ginkel et al., 2003), but in contrast to studies on *S. aureus*, which requires epithelial injury to enter the nerve. From the trigeminal nerve, however, no bacteria were isolated without prior epithelial injury. In contrast, when the olfactory epithelium was injured, we found that *S. agalactiae* entered the trigeminal nerve. This finding suggests that perhaps other cell types restricted to the olfactory neuroepithelium are involved in the mechanism by which *S. agalactiae* enters the olfactory nerve in the absence of injury.

The capacity for intracellular survival in glia has been suggested as a key mechanism by which bacteria can infect the olfactory and trigeminal nerves. Intracellular survival in OECs and/or TgSCs has previously been shown for *S. pneumoniae* (Macedo-Ramos et al., 2014), *C. muridarum* (Nazareth et al., 2021), *B. pseudomallei* (Walkden et al., 2020), and *N. meningitidis* (Delbaz et al., 2020). The current study demonstrates that *S. agalactiae* can survive inside cultured OECs and astrocytes for at least 24 h. The ability to resist killing and to survive in OECs is likely important for olfactory nerve infection, and in astrocytes, for traversing the glia limitans layer and for entry into the CNS (Ruiz-Mendoza et al., 2016). Whilst intracellular survival in OECs has not been previously investigated for *S. agalactiae*, one previous study has shown that *S. agalactiae* (including serotype III) can survive in astrocytes for at least 12 h (Stoner et al., 2015), which is in accordance with our findings. We found that *S. agalactiae* is attenuated for intracellular survival in TgSCs compared to other glial types. This finding was surprising, given the fact that OECs have been shown to mount a much more powerful immune response to *Escherichia coli* and pathogen-associated molecular patterns (PAMPs) (Vincent et al., 2007), as well as exhibit higher levels of immune- and inflammatory-related factors on the transcriptional level (Vincent et al., 2005) than Schwann cells. It is important to note that these previous investigations used Schwann cells from the sciatic nerve, and not the trigeminal nerve; TgSCs may exhibit differential immune responses than other Schwann cells due to their anatomical location near the nasal cavity (Choudhury et al., 2021). A recent study, however, has shown that OECs are better protected against intracellular survival of *C. muridarum* (Nazareth et al., 2021), and are more efficient phagocytes of necrotic cellular debris, than TgSCs (Nazareth et al., 2020). It is thus possible that OECs and TgSCs may respond differently to distinct phagocytic targets and while colocalisation of LAMP2 with the internalised bacteria appeared similar across the different glial types, further investigations may reveal mechanisms that modulate intracellular survival or destruction of the bacteria in the different glia. Whilst the intact nasal epithelium is likely to be the key to protecting the trigeminal nerve from infection, the low capacity for survival in TgSCs may also contribute to the lack of trigeminal nerve *S. agalactiae* infection.

Glial activation is one of the first hallmarks of nervous system infection and neuroinflammation, characterized by cytokine and chemokine release (Refolo and Stefanova, 2019). These factors are protective in that they stimulate clearance of infectious agents and cell debris, with some factors also directly promoting tissue repair. However, if inflammation is excessive and long-lasting, it can lead to neurotoxicity and tissue damage. The host inflammatory response to *S. agalactiae* contributes significantly to the pathogenesis of meningitis and CNS injury (Patras and Nizet, 2018). We here showed that *S. agalactiae* infection resulted in the production of many pro- and anti-inflammatory cytokines and chemokines by glia.

All three glial types responded with secretion of the pro-inflammatory cytokines IL-12, TNF-α, IL-1β, IL-17 and IFN-γ, which are also secreted at high levels in mouse models of *S. agalactiae* sepsis (Patras and Nizet, 2018). IL-12, TNF-α and IL-1β are key mediators of for the initial inflammation cascade in response to bacteria (Wennekamp and Henneke, 2008), including *S. agalactiae* (Patras and Nizet, 2018). IL-12, in particular, has key roles in combatting *S. agalactiae* infections (Patras and Nizet, 2018), including urinary tract infection (Sullivan et al., 2016) and sepsis (Landwehr-Kenzel and Henneke, 2014). High levels of TNF-α can cause damage to the brain tissue, but peripheral glia produce pituitary adenylate cyclase activating peptide (PACAP) (Hegg et al., 2003), which may protect against TNF-α-mediated damage (Kanekar et al., 2010). Production of pro-inflammatory cytokines was overall highest in OECs, followed by TgSCs and then astrocytes. Secretion of these factors may be important for OECs and TgSCs (peripheral glia) in preventing microbial invasion of the brain *via* peripheral nerves.

The pro-inflammatory glial responses were counter-balanced by the generation of the anti-inflammatory cytokines IL-6 and IL-10. IL-6 is important for regeneration/cell survival, whereas IL-10 overall limits inflammation (Landwehr-Kenzel and Henneke, 2014). Astrocytes induce transcription of IL-6 in response to *S. agalactiae* infection (Stoner et al., 2015), which is considered important for prevention of *S. agalactiae*-induced damage to the CNS tissue (Landwehr-Kenzel and Henneke, 2014). OECs and TgSCs have previously been shown to produce IL-12 and IL-6 in response to both Gram-negative and Gram-positive bacteria (Dando et al., 2016; Choudhury et al., 2021; Nazareth et al., 2021). Thus, secretion of IL-12 and IL-6 may be key mediators of the peripheral glial responses to a wide range of infectious agents. In the current study, we found that the glia also responded to *S. agalactiae* with secretion of several chemokines (usually secreted secondary to pro-inflammatory cytokines) that mediate recruitment of leukocytes (Graves and Jiang, 1995) and have important roles in neuroinflammation and neural repair (Miller et al., 2008).

*S. agalactiae* produces a polysaccharide capsule, which protects against opsonisation and, thus, opsonophagocytosis by several cell types, as well as suppresses oxidative burst and subsequent release of proteases by neutrophils (Carlin et al., 2009). *S. agalactiae* are classified into 10 different serotypes based on capsule composition (Afshar et al., 2011), however, 0.5% of clinical *S. agalactiae* isolates do not react with any diagnostic anti-capsular serum (Ippolito et al., 2010), and are considered non-typeable (NT). NT strains may either completely or in part lack the capsule. Human clinical isolates completely lacking the entire capsular locus have been identified using PCR (Creti et al., 2012). In a murine model of urinary tract infection, lack of the *S. agalactiae* type III capsule was shown to enhance bladder colonization (Sullivan et al., 2016).

In the current study, we showed that the capsule did not affect adhesion and intracellular survival by *S. agalactiae* in OECs and astrocytes. These findings suggest that capsule-independent mechanisms mediate attachment and internalization of *S. agalactiae* in these cells, as has previously been shown for dendritic cells in which both capsule-dependent and independent internalization mechanisms have been described (Lemire et al., 2012a). Regarding astrocytes, this finding contradicts a previous study, which showed that presence of the capsule impaired intracellular survival of *S. agalactiae*, however the study used *S. agalactiae* serotype I and not III (Alkuwaity et al., 2012). In TgSCs, however, intracellular survival of wild-type *S. agalactiae* was negligible, whereas the capsule-deficient mutant exhibited significant intracellular survival (in fact, higher than in astrocytes); thus, the capsule appeared to repress intracellular survival in TgSCs, however the mechanism is yet to be determined. It is worth noting that our assays used heat-inactivated bovine serum (inactivated complement system) in the medium; this would reduce *S. agalactiae* capsule interaction with the opsonizing effects of the complement system (Maisey et al., 2008; Patras and Nizet, 2018).

We found that the capsule also moderately affected the production of cyto/chemokines. Overall, the presence of the capsule repressed the production of multiple cyto/chemokines by the glia, but enhanced secretion of some specific ones by OECs. Overall, the capsule promoted secretion of some chemokines by OECs, whilst having the opposite effects on TgSCs and astrocytes. Thus, the interrelationship between cytokine production and intracellular survival remains unclear.

The study has several limitations, which are important to address in future investigations of how *S. agalactiae* invade the CNS *via* cranial nerves and infect glia. Long-term *in vivo* studies are required to determine *S. agalactiae* survival in the nerves and CNS, and whether *S. agalactiae* causes associated neuropathologies as has been shown for *C. pneumoniae* and HSV-1 (Balin et al., 2018) for example. The cell cultures had purities of 70-80% and thus the cytokine responses reflect the total cell population and not just the target glial cells; organoid type 3D cultures in future may reveal more *in vivo* like responses. Further investigations focussing on the potential for *S. agalactiae* to cause morphological changes, such as nuclear abnormalities, are also needed.

## Conclusion

In the current study, *S. agalactiae* was shown to invade the CNS *via* the olfactory nerve in mice after intranasal inoculation. We also showed that epithelial injury was associated with increased infection of the olfactory nerve and also led to *S. agalactiae* infection of the trigeminal nerve. Finally, we showed that *S. agalactiae* also infected and survived intracellularly in the glia of both the olfactory and the trigeminal nerves and CNS. Importantly, this ability to infect glia may also constitute a key reason for the bacterial invasion of these cranial nerves and the brain.

## Data Availability Statement

The original contributions presented in the study are included in the article/**Supplementary Material**. Further inquiries can be directed to the corresponding author.

## Ethics Statement

All procedures were approved by the Griffith University Biosafety Committee (NLRD/09/15_var7) and the Griffith University Animal Ethics Committee (MSC/08/18/AEC) in accordance with guidelines of the Australian Commonwealth Office of Gene Technology Regulator and the National Health and Medical Research Council of Australia.

## Author Contributions

Experiments were conducted by AC, AD, IC, TE, MS, and CG. Figures were prepared by AC, AD, JS, and JE. All authors analysed the data. AC, AD, and JE wrote the main manuscript and all authors reviewed and edited the manuscript. JE and GU provided the overall supervision of the project. All authors contributed to the article and approved the submitted version.

## Funding

This study was supported by a Menzies Health Institute Queensland Capacity Grant (Griffith University) to JE, MS, and GU and a Clem Jones Foundation grant to JE and JS. The funders had no role in study design, data collection and interpretation, or the decision to submit the work for publication.

## Conflict of Interest

The authors declare that the research was conducted in the absence of any commercial or financial relationships that could be construed as a potential conflict of interest.

## Publisher’s Note

All claims expressed in this article are solely those of the authors and do not necessarily represent those of their affiliated organizations, or those of the publisher, the editors and the reviewers. Any product that may be evaluated in this article, or claim that may be made by its manufacturer, is not guaranteed or endorsed by the publisher.
