# Supplementary material for: Streptococcus agalactiae Infects Glial Cells and Invades the Central Nervous System via the Olfactory and Trigeminal Nerves
Source: Front Cell Infect Microbiol. 2022 Feb 24;12:793416. doi: 10.3389/fcimb.2022.793416 (PMC8907725; doi:10.3389/fcimb.2022.793416)
Supplement: Supplementary file 1 [file DataSheet_1.pdf]

# *Streptococcus agalactiae* infects glial cells and invades the central nervous system *via* the olfactory and trigeminal nerves

**Anu Chacko, Ali Delbaz, Indra N. Choudhury, Tanja Eindorf, Megha Shah, Christopher Godfrey, Mathew J. Sullivan, James A. St John, Glen C. Ulett, Jenny A.K. Ekberg**

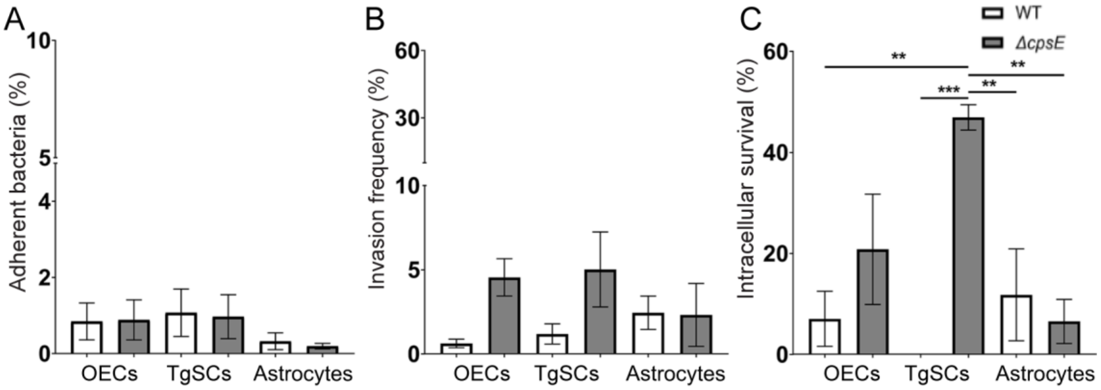


Supplementary Figure 1. (A) The percentage of adherent bacteria at 1 h relative to the initial inoculum. (B) The percentage of invading bacteria was determined by comparing the bacterial load of intracellular bacteria at 2 h with adherent bacteria present at 1 h. (C) The percentage of surviving bacteria was then calculated by comparing intracellular survival at 24 h with survival at 2 h. Data shows mean ± SEM of three independent experiments with n = 3 technical replicates. Data were compared between glial types using two-way ANOVA with Tukey's multiple comparison test; $^{**}p \leq 0.01$, $^{***}p \leq 0.001$.

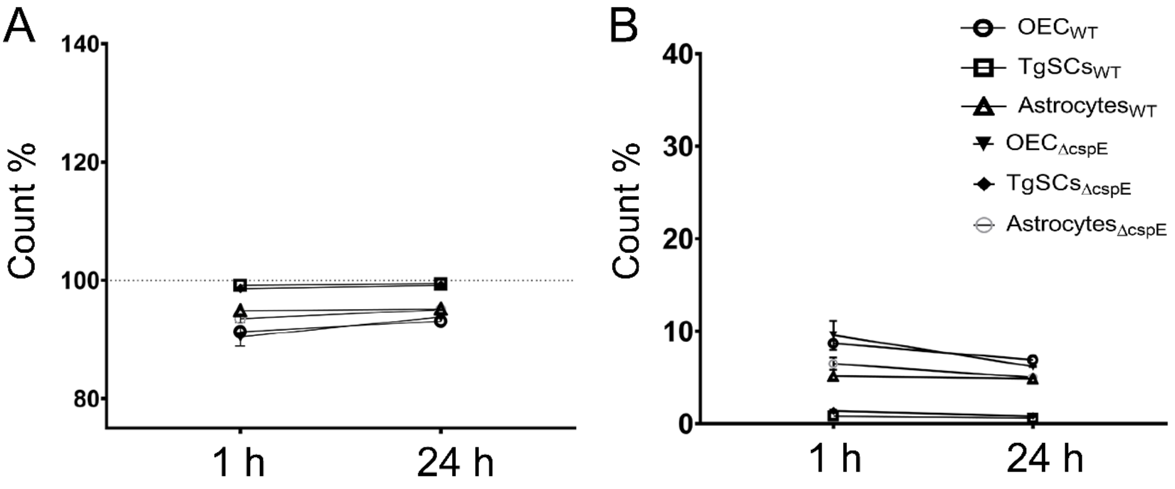


Supplementary Figure 2. *In vitro* glial cells cytotoxicity at 1 h and 24 h post infection (A) Percentage of total cells (B) Percentage of dead cells. Data shows mean ± SEM of three independent experiments with n = 3 technical replicates. Data were compared using two-way ANOVA with Tukey's multiple comparison test.

Supplementary Table 1. Detection of *S. agalactiae* in different organs and conditions

| **Organ** | **4h post infection** | **24h post infection** |
|---|---|---|
| | ***PBS only control*** | |
| Olfactory mucosa | Not detected | Not detected |
| Olfactory bulb | Not detected | Not detected |
| Trigeminal nerve | Not detected | Not detected |
| Brain | Not detected | Not detected |
| Lung | Not detected | Not detected |
| Liver | Not detected | Not detected |
| Blood | Not detected | Not detected |
| | ***Methimazole only control*** | |
| Olfactory mucosa | Not detected | Not detected |
| Olfactory bulb | Not detected | Not detected |
| Trigeminal nerve | Not detected | Not detected |
| Brain | Not detected | Not detected |
| Lung | Not detected | Not detected |
| Liver | Not detected | Not detected |
| Blood | Not detected | Not detected |
| | ***S. agalactiae infection*** | |
| Lung | Not detected | Not detected |
| Liver | Not detected | Not detected |
| Blood | Not detected | Not detected |
| | ***Methimazole + S. agalactiae infection*** | |
| Lung | Not detected | Not detected |
| Liver | Not detected | Not detected |
| Blood | Not detected | Not detected |

Supplementary video 1. Three-dimensional reconstruction of a confocal z-stack of *S. agalactiae* in phagolysosomes within OECs. Nucleus (cyan), *S. agalactiae* (green), Lamp-2 (magenta), OEC (red).

Supplementary video 2. Three-dimensional reconstruction of a confocal z-stack of *S. agalactiae* in phagolysosomes within TgSCs. Nucleus (cyan), *S. agalactiae* (green), Lamp-2 (magenta), TgSC (red).

Supplementary video 3. Three-dimensional reconstruction of a confocal z-stack of *S. agalactiae* in phagolysosomes within astrocytes. Nucleus (cyan), *S. agalactiae* (green), Lamp-2 (magenta), astrocyte (red).
